# Broadband Piezoelectric Energy Harvester Based on Coupling Resonance Frequency Tuning

**DOI:** 10.3390/mi14010105

**Published:** 2022-12-30

**Authors:** Kun Hu, Min Wang

**Affiliations:** 1 Department of Mechanics and Aerospace Engineering, Southern University of Science and Technology, Shenzhen 518055, China; 2 School of Microelectronics, Southern University of Science and Technology, Shenzhen 518055, China

**Keywords:** piezoelectric energy harvester, broadband, coupling, frequency tuning, electrode coverage

## Abstract

The bandwidth of piezoelectric energy harvesters (PEHs) can be broadened by resonance-based frequency tuning approaches, including mechanical tuning and electrical tuning. In this work, a new coupling tuning mechanism for regulating the near-open-circuit resonance frequency by changing the effective electrode coverage (EEC) is presented. A linear model of a bimorph piezoelectric cantilever with segmented electrodes is used to evaluate the power harvesting behavior near the open-circuit resonance frequency when EEC changes from 0 to 100%. According to the theoretical analysis, it is found that the variation of EEC brings about the change in coupling strength, which is positively associated with the near-open-circuit resonance frequency of PEH. Two cantilever PEHs with segmented electrodes based on PZT and PZT-PT are constructed for validation of the coupling tuning mechanism. The analytical and experimental results illustrate remarkable improvements in both bandwidth and average power through the coupling resonance frequency tuning method. In addition, adopting extraordinary piezoelectric single crystals and optimizing the proof mass and piezoelectric layer dimensions were theoretically shown to be effective methods for further improvement of bandwidth.

## 1. Introduction

In the last two decades, energy harvesting from the natural environment has received great attention for the prospects of powering portable electronics and low-power consumption devices in wireless sensor network applications [1,2,3,4]. Among different energy extraction methods, vibrational piezoelectric energy harvesters (PEHs) are widely used due to their high energy density, the feasibility of fabrication, and ease of integration [5,6,7]. For the commonly employed linear PEHs, substantial power can be only obtained within the bandwidth near the resonance frequency. When the frequency of excitation deviates slightly from the bandwidth, the output power decreases drastically [8,9]. To achieve high power generation over a larger, continuous bandwidth for certain conditions, various bandwidth widening approaches, including nonlinear mechanism [10,11,12], multi-degree-of-freedom mechanism [13], frequency-up-conversion mechanism [14], and hybrid harvesting mechanism [15], have been adopted. Compared to bandwidth widening, the frequency tuning approaches are more generic solutions for various excitations and have the capability to widen the bandwidth over larger ranges [5,16,17,18]. The mechanical frequency tuning method has been used to adjust the effective stiffness of the vibration system by applying additional magnetic [19] or piezoelectric force [20] to achieve resonance frequency change. However, the additional actuators bring about quite a little increase in both volume and weight, which results in a decrease in relative power density. The electrical frequency tuning method is normally realized by adjusting the interface circuit to change the equivalent independence of the load [21]. Whereas the average output power is relatively low compared to the overall maximum power because the impedance matching is less than optimal in the effective frequency range. In addition, for either electrical or mechanical tuning methods, simplifying the system and minimizing the extracted power consumption of tuning actuators are still challenging for practical applications. For cantilever PEHs, the near-open-circuit resonance frequency has been theoretically shown to be related to the coupling strength [22]. Furthermore, effective electrode coverage (EEC) can affect the output power characteristics dramatically by altering the coupling strength of PEH [23,24,25,26]. Thus, in this paper, we propose a broadband energy harvester with a coupling resonance frequency tuning mechanism by exploiting variable EEC.

## 2. Coupling Resonance Frequency Tuning Mechanism

### 2.1. Modeling

**Figure 1:**
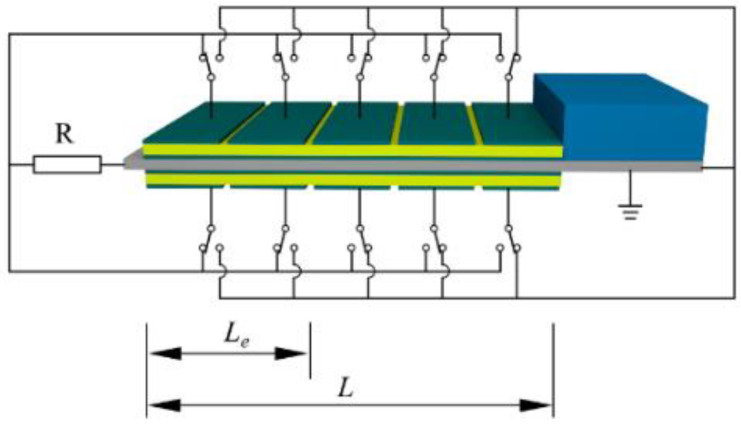
Schematic diagram of the bimorph cantilever with segmented electrodes and switchable electrical connections.

As shown in Figure 1, the proposed piezoelectric energy harvester is a bimorph cantilever with a tip-proof mass. Two piezoelectric layers are symmetrically bonded onto the central metal substrate. The metallic substrate serves as the common grounding electrode, so electrical parallel connections are formed between piezoelectric layers. The outer electrodes of piezoelectric layers are divided into uniform segments. Each segmented electrode connects a single-pole double-throw switch. The segmented electrodes are only effective for output power when connected to the load resistance. The effective electrode coverage (EEC) is defined as *r_e_* = *L_e_*/*L* × 100%, where *L_e_* is the total length of the effective electrodes, and *L* is the total length of the piezoelectric layer.

When excited by weak vibrations, a piezoelectric cantilever can be simplified as a linear system. For harmonic base excitation of pure base translation, the base motion can be expressed in the form of *Ae^jwt^*, where *A* and *ω* are the amplitude of acceleration and the angular frequency of base excitation, respectively. Following the linear system assumption for cantilevered harvesters, the output voltage and the *r*th order modal response are also harmonic and can be expressed in the form of *v*(*t*) = *Ve^jwt^* and *ηr* = *H_r_e^jwt^*, respectively, where *V* and *H_r_* are the corresponding amplitudes. The modal electromechanical equations of the piezoelectric cantilever with variable electrode coverage can be expressed as [27]

(1) $$\left(\omega_{r}^{2}-\omega^{2}+j2\zeta_{r}\omega_{r}\omega \right)H_{r}-{\bar{\theta }}_{r}V={\bar{M}}_{r}A\;\;\;\;r=1,2,3\cdots$$

(2) $$\left(\frac{1}{R}+j\omega C_{eq}\right)V+j\omega \overset{\infty }{\underset{r=1}{\sum }}{\bar{\theta }}_{r}H_{r}=0$$

where ${\bar{\theta }}_{r}\;$is the *r*th-order piezoelectric coupling factor, which is closely related to the EEC and is given by

(3) $${\bar{\theta }}_{r}=e_{31}b\left(h_{p}+h_{s}\right)\phi_{r}^{\prime }\left(L_{e}\right)$$

where *e*_31_ is the effective piezoelectric stress constant, *b* is the width of the beam, *h_s_* and *h_p_* are the thicknesses of the substrate and piezoelectric layer, respectively. *ϕ_r_*(*x*) is the mass-normalized eigenfunction of the *r*th-order vibration mode, which can be determined with the boundary conditions and the orthogonality conditions. In addition, *ζ_r_* is the *r*th-order modal mechanical damping ratio that includes the effects of both strain rate damping and viscous air damping, ${\bar{M}}_{r}$ is the *r*th-order effective modal inertia force, *R* is the load resistance, and *C_eq_* is the equivalent capacitance of the effective piezoelectric units. The detailed expressions of ${\bar{M}}_{r}$ and *C_eq_* are given in Appendix A.

Substituting Equation (1) into (2), *V* and *H_r_* can be obtained explicitly, and *v*(*t*) can be expressed as

(4) $$v\left(t\right)=e^{j\omega t}\frac{\sum_{r=1}^{\infty }\frac{j\omega \gamma_{r}{\bar{M}}_{r}A}{\omega_{r}^{2}-\omega^{2}+j2\zeta_{r}\omega_{r}\omega }}{\frac{1}{R}+j\omega C_{eq}+\sum_{r=1}^{\infty }\frac{j\omega {\bar{\theta }}_{r}^{2}}{\omega_{r}^{2}-\omega^{2}+j2\zeta_{r}\omega_{r}\omega }}$$

To obtain the maximum output power, energy harvesters are mostly used to work near the 1st resonance frequency. When the driving frequency *ω* is near the fundamental resonant frequency *ω*_1_, the voltage response can reduce to the first order component [28], and the power delivered to the connected load is calculated as

(5) $$P_{rms}=\frac{{\left(\bar{M}A\right)}^{2}}{2\omega_{1}}\times \frac{k_{e}^{2}{\bar{\omega }}^{2}\bar{R}}{{\left[1-{\bar{\omega }}^{2}\left(1+2\zeta_{1}\bar{R}\right)\right]}^{2}+{\left[2\zeta_{1}\bar{\omega }+\bar{R}\bar{\omega }\left(1-{\bar{\omega }}^{2}+k_{e}^{2}\right)\right]}^{2}}$$

where $\bar{\omega }$ is the dimensionless angular frequency and expressed as $\bar{\omega }=\frac{\omega }{\omega_{1}},\;\bar{R}$ is the dimensionless load resistance and expressed as $\bar{R}=\omega_{1}RC_{eq}$, $k_{e}^{2}$ is the first-order effective electromechanical coefficient and expressed as $k_{e}^{2}={\bar{\theta }}_{1}^{2}/\left(C_{eq}\omega_{1}^{2}\right)$.

For strong electromechanical coupling harvesters, two maximum power peaks can be obtained on different optimal resistances at two particular excitation frequencies, one near the short-circuit resonance (NSCR) frequency and the other near the open-circuit anti-resonance (NOCR) frequency [28]. The latter can be given by

(6) $${\bar{\omega }}_{\mathrm{noc}}=\sqrt{\left(1+\frac{k_{e}^{2}}{2}-2\zeta_{1}^{2}\right)+\sqrt{{\left(\frac{k_{e}^{2}}{2}-2\zeta_{1}^{2}\right)}^{2}-4\zeta_{1}^{2}}}$$

Because the mechanical damping ratio is always less than 0.1, the dimensionless NOCR frequency can be approximated to the open-circuit resonance frequency and expressed as

(7) $${\bar{\omega }}_{\mathrm{oc}}=\sqrt{1+k_{e}^{2}}$$

### 2.2. Frequency Broadening Mechanism

**Figure 2:**
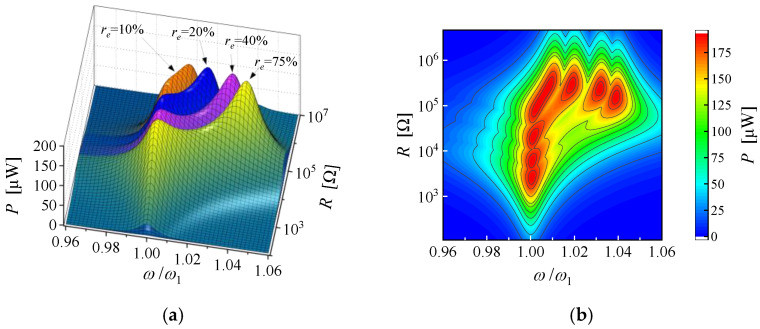
(**a**) The distribution of the individual output power picks and (**b**) the contour map of the overall maximum power of the bimorph cantilever with a proof mass for EEC of 10%, 20%, 40%, and 75%.

According to Equation (7), the NOCR frequency of the piezoelectric cantilever has a positive relationship with the effective electromechanical coefficient. Furthermore, the electromechanical coefficient can be regulated by adjusting the EEC [23]. Therefore, the NOCR frequency is promising to be tuned by switching the connections of the piezoelectric electrodes. The theoretical output power frequency response functions (FRFs) near the fundamental resonance frequency of a bimorph cantilever at different EECs of 10%, 20%, 40% and 75% are shown in Figure 2. The detailed structural and material parameters of the cantilever are listed in Table A1 (Appendix A). The base acceleration is 2 m/s^2^, and the mechanical damping ratio is assumed to be 0.005. Figure 2a illustrates the distribution of the power FRF resonance picks, and Figure 2b shows the contour map of the overall maximum power of four EECs conditions. In these four EEC conditions, the PEH always behaves as a strongly coupled system and has two maximum power FRFs picks. All the NSCR picks, and NOCR picks have the same maximum power FRF of 192.3 μW. With the increase of EEC, the frequency of the NSCR pick almost remains constant while the corresponding optimal load resistance gradually increases. However, the frequency of the NOCR picks increases with the EEC, while the optimal resistance loads change slightly. When the EECs are 10%, 20%, 40% and 75%, the nondimensionalized NOCR frequencies are 1.008, 1.019, 1.032 and 1.039, respectively, and the optimal load resistances are 245.6 kΩ, 298.6 kΩ, 233.9 kΩ and 150.8 kΩ, respectively. The elliptic form of the contour lines of NOCR picks also depict that the output power shows weak sensitivity to the variations in the load resistor.

**Figure 3:**
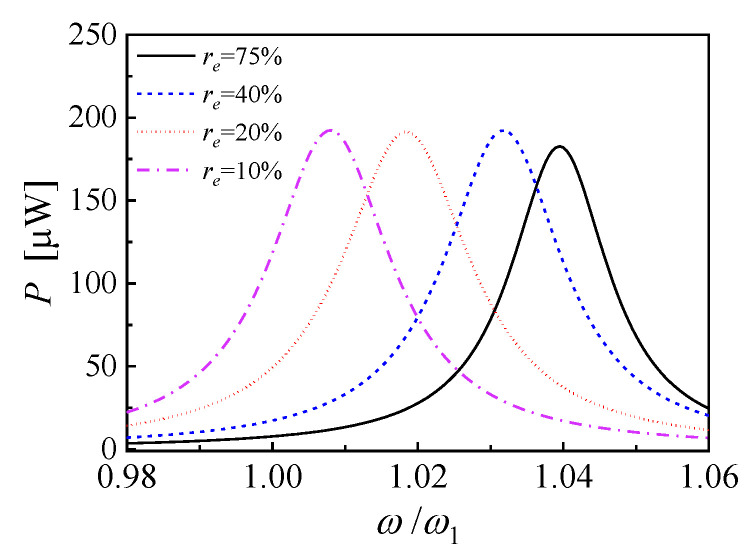
The power frequency response curves at the load resistance of 245.6 kΩ with EEC of 10%, 20%, 40%, and 75%.

Figure 3 shows a slice map of Figure 2a at the load resistance of 245.6 kΩ. The power frequency response curves connect and overlap with each other, and a wider built-up bandwidth range of high output power is obtained. Table 1 further shows the optimal load resistance, the independent half power bandwidth and the corresponding average power FRF in the bandwidth range for each EEC. Compared to the maximum bandwidth of 2.1% and maximum effective average power of 150.2 μW of individual EEC conditions, a wider frequency bandwidth of 4.9% and a higher effective average power of 164.4 μW can be obtained by employing coupling resonance frequency tuning. Therefore, in this approach, the resonance frequency can be tuned by changing the EEC of a single piezoelectric cantilever without any mechanical actuator. The switchover of the electrical connections can be realized with a CMOS analog switch, which is easy to be integrated and consumes only minimal energy. In addition, the PEH always works in quasi-optimal impedance matching conditions.

## 3. Influencing Factors of Frequency Modulation Bandwidth

### 3.1. Effective Electromechanical Coefficient 

**Figure 4:**
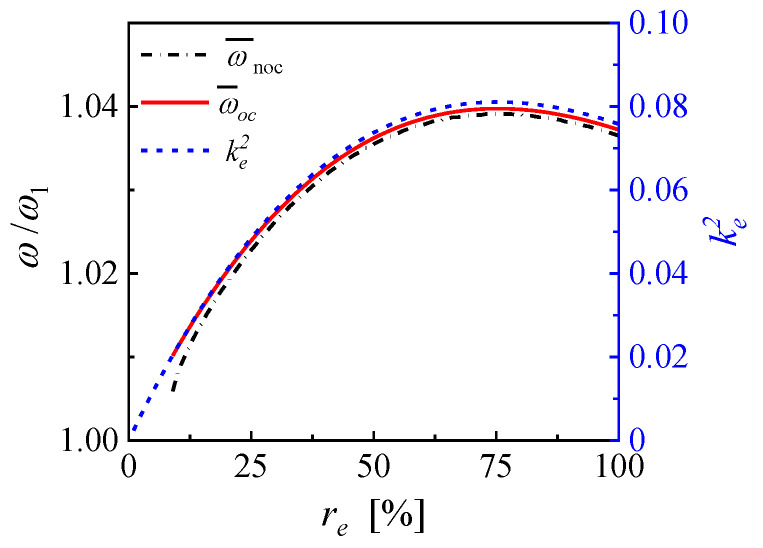
The variations of the NOCR frequency, the open-circuit resonance frequency, and the effective electromechanical coupling coefficient versus EEC.

With sufficient subdivisions of the electrode, the overlapping and connections of individual output power response curves of different EECs can always be obtained. The overall frequency bandwidth mainly depends on the maximum frequency shift of the NOCR frequency away from the fundamental resonance frequency. Thus, the available bandwidth is significantly extended over a wider interval of the frequency spectrum. According to Equations (6) and (7), the NOCR frequency can be approximated in the open-circuit resonance frequency and is closely related to the effective electromechanical coefficient. It is seen that a higher effective electromechanical coefficient results in the wider frequency bandwidth. When the mechanical damping ratio is 0.005, the variations of the NOCR frequency, the open-circuit resonance frequency, and the effective electromechanical coupling coefficient versus the effective electrode coverage are shown in Figure 4. All $k_{e}^{2}$, ${\bar{\omega }}_{\mathrm{oc}}$, and ${\bar{\omega }}_{\mathrm{noc}}$ increase at the beginning, then reach a maximum value, and finally drop slightly with the EEC. Because the critical electromechanical coupling coefficient is 0.0201, ${\bar{\omega }}_{\mathrm{noc}}$ is only valid when the EEC is no less than 8.9% so that the harvester is strongly coupled. When the EEC is 8.9%, $k_{e}^{2}$ is 0.0202, ${\bar{\omega }}_{\mathrm{oc}}$ is 1.010, ${\bar{\omega }}_{\mathrm{noc}}$ is 1.005, and the optimal load resistance is 223.2 kΩ. When the EEC is 75.3%, $k_{e}^{2}$ reaches the maximum value of 0.0811, ${\bar{\omega }}_{\mathrm{oc}}$ is 1.0398, ${\bar{\omega }}_{\mathrm{noc}}$ is 1.0391, and the optimal load resistance is 153.6 kΩ. Therefore, ${\bar{\omega }}_{\mathrm{noc}}$ can be tuned between 1.005 and 1.0391 by adjusting the EEC from 8.9% to 75.3%. Because the variations of ${\bar{\omega }}_{\mathrm{oc}}$ and ${\bar{\omega }}_{\mathrm{noc}}$ are almost the same as that of $k_{e}^{2}$, $k_{e}^{2}$ can be used to estimate the frequency modulation bandwidth.

**Figure 5:**
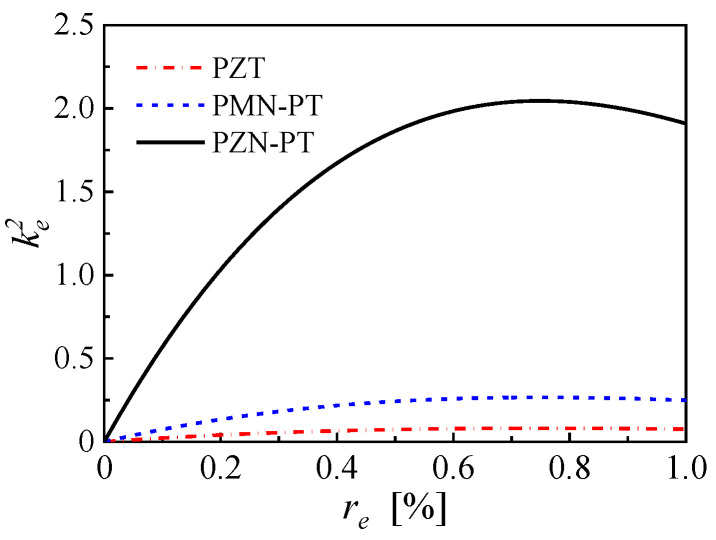
The effective electromechanical coupling coefficients of the bimorph cantilevers with a proof mass and based on PZT, PMN-PT, and PZN-PT individually.

Substituting structure and material parameters into ${\bar{\theta }}_{1}$ and *C_eq_*, the effective electromechanical coefficient can be rewritten as

(8) $$k_{e}^{2}=\frac{\phi_{1}^{\prime }{\left(r_{e}L\right)}^{2}}{r_{e}}\times \frac{e_{31}^{2}}{\varepsilon_{33}^{S}\varepsilon_{0}}\times \frac{bh_{p}{\left(h_{p}+h_{s}\right)}^{2}}{2\omega_{1}^{2}L}$$

where the first term on the right-hand side is strongly related to the EEC, the second term is related to the piezoelectric properties of the piezoelectric layer, and the third term is determined by the basic structure and the mechanical properties of the cantilever. In addition, the second term of Equation (8) directly relates to the piezoelectric, elastic and dielectric properties of the material and characterizes the effectiveness of converting the mechanical energy into electrical energy, which is similar to the expression of electromechanical coupling factor $K_{31}^{2}$. Compared to traditional PZT, the PMN-PT and PZN-PT (the solid solution between Lead titanate and Lead magnesium niobate or Lead zinc niobate) single crystals have been found to have extraordinary electromechanical coupling factors and are applied into various fields such as sensors, actuators and sonar transducers [29]. The properties comparison of PZT, PMN-PT, and PZN-PT is shown in Table A2. With the same structure given by Table A1, the variations of the effective electromechanical coupling coefficients versus EEC for bimorph cantilevers based on PZT, PMN-PT, and PZN-PT are shown in Figure 5.

The effective electromechanical coupling coefficients of the PEHs based on PZT, PMN-PT, and PZN-PT achieve the maximum values of 0.0811 at EEC of 75.3%, 0.2664 at EEC of 75%, 2.0452 at EEC of 74.9%, respectively. The slight difference in the EECs that take the maximum value is mainly due to the diversity of elastic constants and the density of piezoelectric materials. Meanwhile, own to the extraordinary piezoelectricity, the maximum effective electromechanical coupling coefficient is obtained by the PEH based on PZT-PT, which is more than 25 times as much as that of PZT and almost 8 times as much as that of PMN-PT.

**Figure 6:**
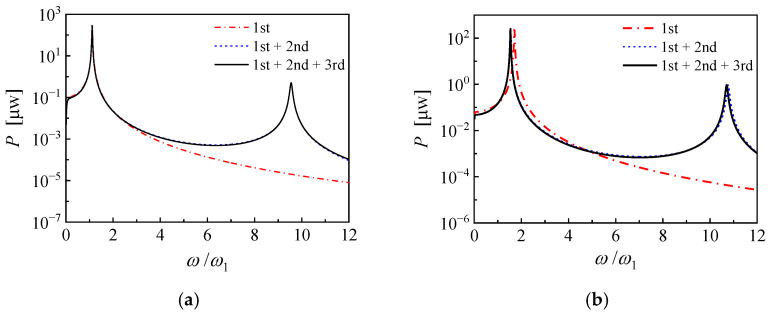
Comparisons of the unimodal and the multi-modal power frequency responses of the piezoelectric cantilevers with a proof mass based on (**a**) PMN-PT and (**b**) PZN-PT.

According to the above results obtained from unimodal solution, the PEHs based on PZN-PT are promising to gain a noteworthy frequency shift of NOCR frequency. However, when the excitation frequency deviates away from the resonance frequency of cantilever PEHs, the approximation accuracy of the unimodal solution is no longer sufficient. Comparisons of the unimodal and the multi-modal power frequency responses of the piezoelectric cantilevers based on PMN-PT and PZN-PT are shown in Figure 6. The EEC is invariable 100%, the base acceleration is 2 m/s^2^, and the mechanical damping ratio is 0.005. It is seen that the solution comprising the first two vibration modes agrees well with the solution comprising the first three vibration modes when the operating frequency is below the second-order natural frequency. Therefore, the solution comprising the first two vibration modes is a valid approximation for output power evaluation in this frequency range. For two PEHs, both the NOCR frequency and the corresponding maximum power obtained from unimodal solution deviate from those obtained from multi-modal solution in different degrees. Table 2 shows the detailed NOCR frequencies, maximum powers, and the corresponding optimal load resistances of the two PEHs. The unimodal solution overestimates the NOCR frequency and the optimal load resistance but underestimates the maximum power. Furthermore, the discrepancies between unimodal solution and multi-modal solution increase with the coupling strength. For the PEH based on PMN-PT, the frequency shifts obtained from unimodal solution and multi-model are 11.7% and 11.2%, respectively. For the PEH based on PZN-PT, the frequency shifts obtained from unimodal solution and multi-modal are 70.5% and 53.6%, respectively. Therefore, it is necessary to evaluate whether the effective electromechanical coupling coefficient obtained from the unimodal solution is still efficient for the estimation of the NOCR frequency shift of ultra-strongly coupled PEHs.

**Figure 7:**
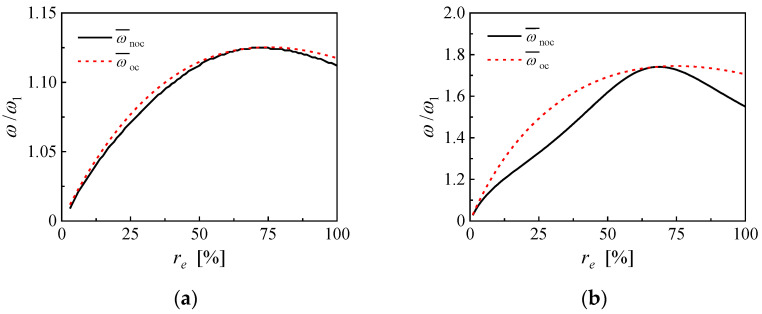
The multi-modal NOCR frequency and the unimodal open-circuit resonance frequency of the PEHs based on (**a**) PMN-PT and (**b**) PZN-PT.

Further comparisons of the NOCR frequency obtained from a multi-modal solution and the open-circuit resonance frequency calculated with the unimodal effective electromechanical coupling coefficient are shown in Figure 7. For PEH based on PMN-PT, the unimodal open-circuit resonance frequency gets the maximum value of 1.125 at an EEC of 75%, while the multi-modal NOCR frequency gets the maximum value of 1.125 at an EEC of 72%. For PEH based on PZN-PT, the unimodal open-circuit resonance frequency gets the maximum value of 1.745 at EEC of 75%, while the multi-modal NOCR frequency gets the maximum value of 1.741 at EEC of 69%. Thus, the unimodal open-circuit resonance frequency can still be used to approximate the variation and the maximum value of multi-modal NOCR frequency.

### 3.2. Piezoelectric Property

**Figure 8:**
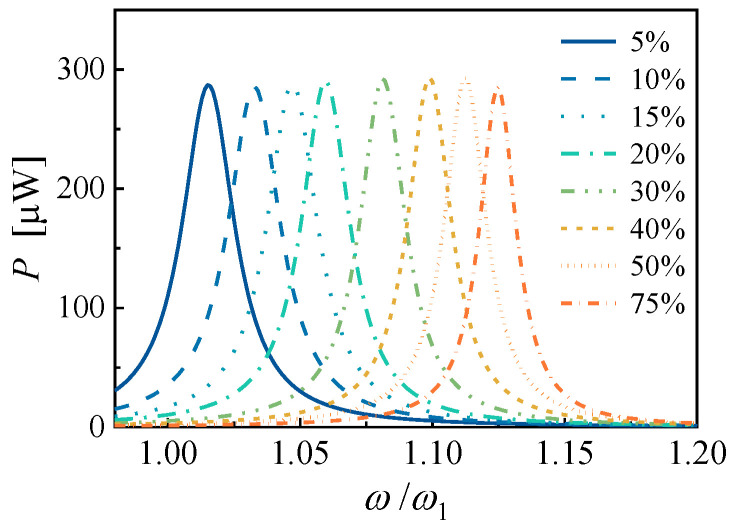
The theoretical output power frequency responses of the linear PMN-PT based piezoelectric cantilever with a proof mass.

According to the effective electromechanical coupling coefficients shown in Figure 5, PEHs fabricated with PMN-PT and PZN-PT are promising to acquire wider frequency modulation bandwidth compared to that of PZT. With the previous structure given by Table A1, the output power FRFs of a PMN-PT based PEH with different EECs to a load resistance of 0.917 MΩ are shown in Figure 8. The base acceleration is also 2 m/s^2^, and the mechanical damping ratio is 0.005. When the EEC of the PEH is switched to 5%, the dimensionless resonance frequency is 1.015 and the maximum output power FRF is 286.9 μW. When the EEC of the PEH is switched to 75%, the dimensionless resonance frequency is 1.125 and the maximum output power FRF is 285.0 μW. The output power FRFs curves overlap with each other, and an overall frequency bandwidth of 12.8% is obtained, which is increased by 161% compared to the PEH based on PZT.

**Figure 9:**
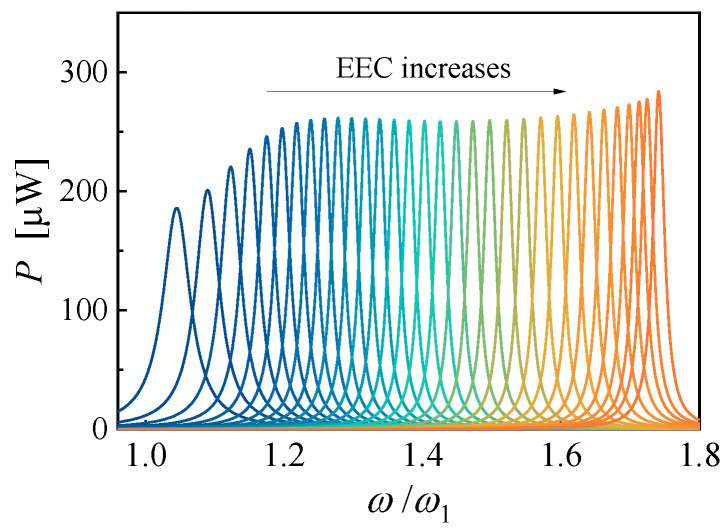
The theoretical output power FRF curves of the PZN-PT based PEH with various EECs.

Similarly, the output power FRFs of a PZN-PT-based PEH with EECs ranging from 2% to 68% with an interval of 2% are shown in Figure 9. The base acceleration is also 2 m/s^2^, the mechanical damping ratio is 0.005, and the load resistance is 6.15 MΩ. When the EEC is switched to 2%, the dimensionless resonance frequency and the maximum power are 1.045 and 186.0 μW, respectively. When the EEC is switched to 68%, the dimensionless resonance frequency and the maximum power are 1.741 and 284.0 μW, respectively. A built-up frequency bandwidth of 71.9% can be obtained, which is more than fourteen times as much as that of PZT-based PEH.

### 3.3. Cantilever Structure Parameter

**Figure 10:**
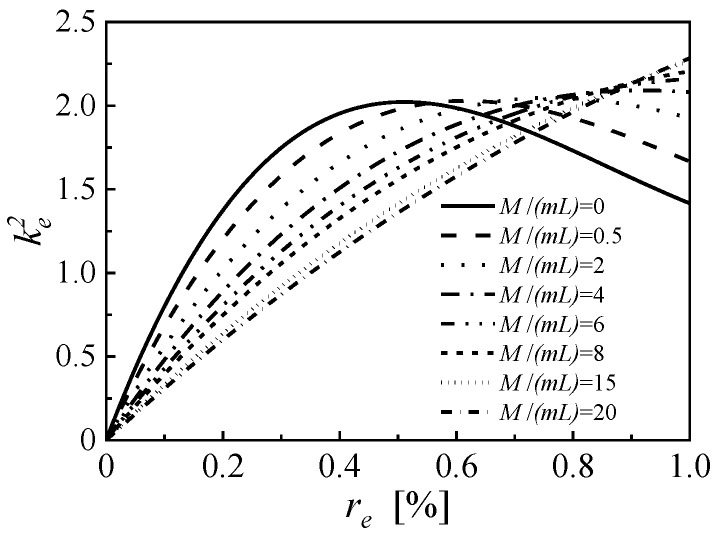
The effective electromechanical coupling coefficients of PZN-PT based PEHs with various proof mass ratios.

**Figure 11:**
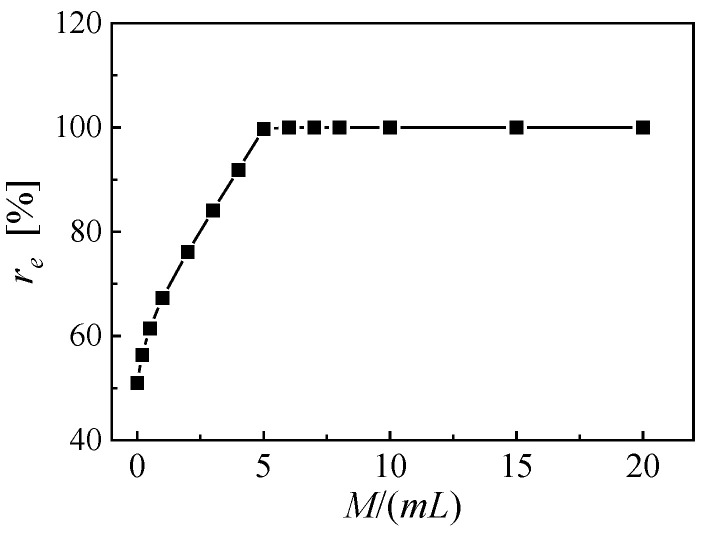
The EEC for taking the maximum effective electromechanical coupling coefficient for PZN-PT based PEHs with various proof-to-mass ratios.

Alongside with the material properties, the third term of Equation (8) directly relates to the laminated structure parameters of the cantilever, including the thicknesses of piezoelectric and substrate layers and the length of the beam. In addition, the fundamental resonance frequency relates to the proof-to-mass ratio (M/mL). In previous studies, the proof mass has been proven to be a key factor that influences the strain distribution on the cantilever and affects the variation of the effective electromechanical coefficient with EEC [23]. On the basis of the structure given by Table A1, a series of cantilevers based on PZT-PT with a proof-to-mass ratio range from 0 to 20 is constructed by simply adjusting the length of the proof mass. The variations of the effective electromechanical coefficient versus EEC are shown in Figure 10. For a cantilever PEH without proof mass, the effective electromechanical coefficient reaches the maximum value of 2.022 at an EEC of 51.0%. With the increasing proof-to-mass ratio, the EEC for taking the maximum value keeps rising, while the maximum effective electromechanical coefficient remains almost constant. When the proof-to-mass ratio is 6, the effective electromechanical coefficient keeps increasing with EEC and takes a maximum value of 2.160 at EEC of 100%. Further increase of proof-to-mass ratio only brings slight growth of the maximum effective electromechanical coefficient. When the proof-to-mass ratio is 20, the effective electromechanical coefficient gets the maximum value of 2.186 at an EEC of 100%. Figure 11 further shows the EEC for taking the maximum effective electromechanical coupling coefficient for PZN-PT based PEHs with various proof-to-mass ratios. When the proof-to-mass ratio exceeds 5, the effective electromechanical coefficient always gets the maximum value at EEC of 100%. For longitudinal space utilization and the convenience of electrode segmentation, the minimum proof-to-mass ratio that makes the effective electromechanical coefficient taking the maximum value at EEC of 100%, is recommended.

**Figure 12:**
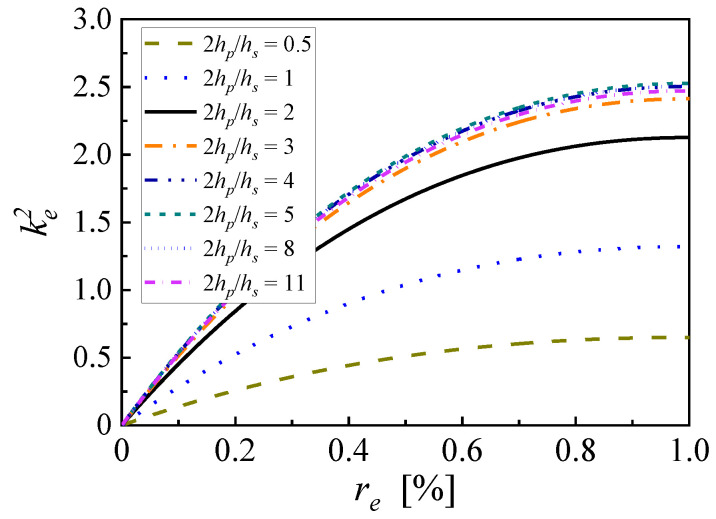
The electromechanical coupling coefficients of PZN-PT based PEHs with various piezoelectric thickness ratios.

The proportion of the piezoelectric material layer along the thickness direction is also a key factor that influences the energy conversion efficiency of PEHs. The electromechanical coupling coefficients of PZN-PT based PEHs with various piezoelectric thickness ratios (2*h_p_*/*h_s_*) are shown in Figure 12. The total thickness of the beam comprising both the substrate and piezoelectric layers is set to 0.6 mm. The proof-to-mass ratio is chosen as 5, with a proof mass length of 26.9 mm. The remaining structural parameters are also given in Table A1. As illustrated above, the effective electromechanical coefficients all take a maximum value at EEC of 100% for different thickness ratios. When the piezoelectric thickness ratio is 0.5, the maximum effective electromechanical coefficient is 0.236. When the piezoelectric thickness ratio increases from 0.5 to 5, the maximum value of the effective electromechanical coefficient keep rising to 2.527, where the thickness of a single piezoelectric layer is 275 μm, and the thickness of the substrate layer is 50 μm. With this condition, the theoretically maximum frequency shift calculated from open-circuit resonance frequency can reach 87.8%. However, a further increase in the piezoelectric thickness ratio leads to a slight decrease in the maximum value of the effective electromechanical coefficient. When the piezoelectric thickness ratio is 11, the maximum effective electromechanical coefficient drops to 2.38. In addition, due to the fragility of most piezoelectric ceramics, an excessive piezoelectric thickness ratio means an unduly thin metal substrate layer that cannot ensure the reliability and stability of PEH devices. Therefore, a moderate piezoelectric thickness ratio is recommended for fabricating broadband piezoelectric energy harvesters.

## 4. Experiment

**Figure 13:**
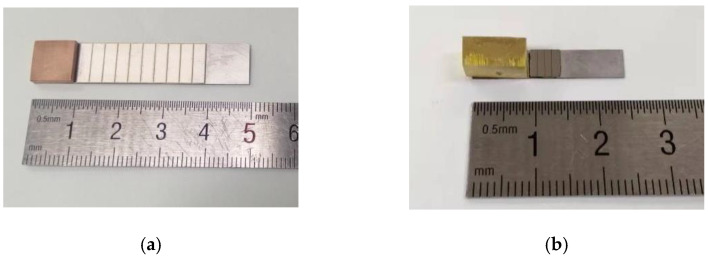
The piezoelectric cantilevers with segmented electrodes and a proof mass based on (**a**) PZT and (**b**) PZN-PT.

To validate the theoretical results, experiments are conducted to obtain the frequency response curves near the first bending modes of two cantilever PEHs based on PZT and PZN-PT, respectively, as shown in Figure 13. As shown in Figure 13a, the PZT based PEH is a bimorph cantilever, and the structure and material details are identical to Table A1. The piezoelectric layers are commercial bulk PZT-5, and the substrate layer is a 304 stainless steel sheet. The outer Ag electrodes on PZT are cut into ten isometric segments by laser for individual connecting to load resistor or common grounding electrode. Therefore, various EECs are obtained by switching electrical connections. As shown in Figure 13b, the PZN-PT-based PEH is a unimorph cantilever with four equal electrodes. The piezoelectric layer is a commercial piezoelectric single crystal (011P-32S, Microfine, Singapore) with a dimension of 5 mm (Length) × 5 mm (Width) × 0.3 mm (Hight). A copper block with a dimension of 10 mm (Length) × 5 mm (Width) × 5 mm (Hight) is chosen as the proof mass.

The experimental setup is the same as the configurations given in [21]. The PEH is clamped in a holder mounted on a shaker (JZK-10, Sinocera, Yangzhou, China) driven by a power amplifier (YE5872A, Sinocera). A variable resistor box is employed as the load resistance. The displacement of the cantilever tip is measured utilizing a laser vibrometer (FNV-R1D-VD1, Holobright, Singapore). The output voltage, acceleration and displacement signals are monitored by an oscilloscope (TDS2024C, Tektronix, Beaverton, Oregon, United States). For the PZT-based PEH, the output voltage on load is directly measured by a passive voltage probe (TPP0201, Tektronix) whose independence is 10 MΩ. For the PZN-PT-based PEH, the output voltage on load is measured by an electrometer (6514 Electrometer, Keithley, Beaverton, Oregon, United States) whose impedance is more than 200 TΩ.

According to preliminary experimental tests and parameters identification, the PEH with EEC of 10% behaves as a weak coupling system and has only one principal power response pick. Nevertheless, the EEC of 10% is chosen as the minimum EEC for the near open-circuit resonance frequency that is close to the fundamental resonance frequency and the local maximum output power that is only slightly lower than the overall maximum output power. In addition, the effective electromechanical coupling coefficient barely changes when the EEC increases from 70% to 80%. Thus, the EEC of 70% is chosen as the maximum EEC to decrease the discrepancy among optimal load resistances of different EECs. To avoid the influence of nonlinear stiffness, damping, and piezoelectricity, the excitation acceleration amplitude is set at 0.5 m/s^2^.

**Figure 14:**
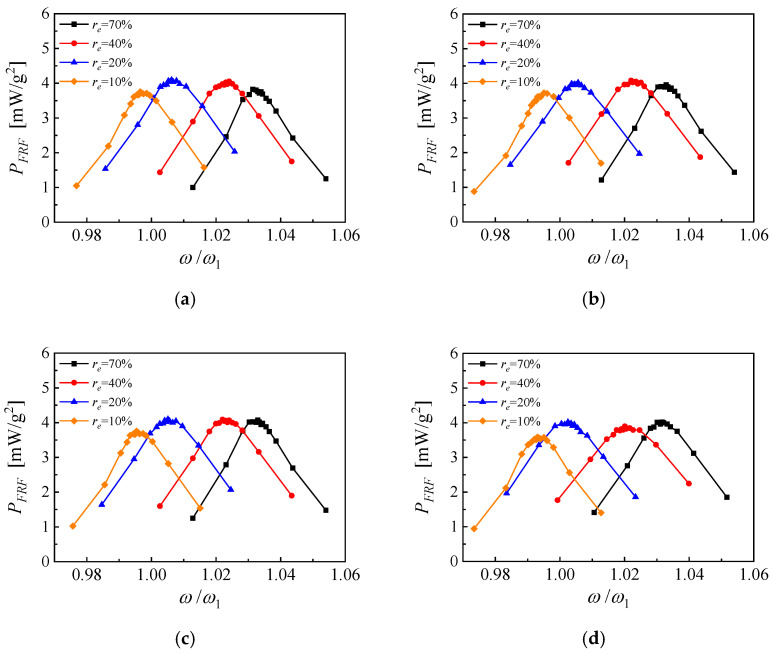
The broadband response obtained by coupling frequency tuning for piezoelectric cantilever based on PZT and with a proof mass (**a**) *R* = 165 kΩ; (**b**) *R* = 131 kΩ; (**c**) *R* = 138 kΩ; (**d**) *R* = 98 kΩ.

For the PEH based on PZT and with EEC of 10%, 20%, 40% and 70%, the measured optimal NOCR load resistances are 165 kΩ, 131 kΩ, 138 kΩ, and 98 kΩ, respectively. The corresponding experimental output power density FRFs of the PEH are shown in Figure 14. When the load resistance is 98 kΩ, the PEH with EEC of 70% has a half-power frequency bandwidth of 3.35%, which is obtained from the linear interpolation of the experimental data. The average power density in the bandwidth range is 3.14 mW/g^2^. With proper adjustment of EEC, a built-up frequency bandwidth of 6.80% and an average power density of 3.44 mW/g^2^ can be obtained, which means an increment of 103% of the bandwidth and an improvement of 9.56% of the average power density are obtained without any additional piezoelectric devices.

**Figure 15:**
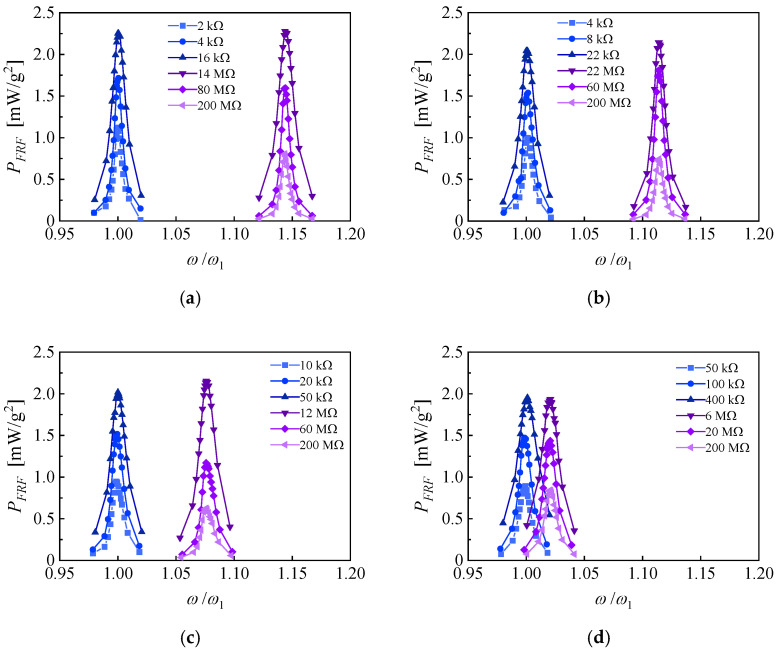
Experimental output power FRFs of the unimorph piezoelectric cantilever based on PZN-PT and with a proof mass (**a**) *r_e_* = 100%; (**b**) *r_e_* = 75%; (**c**) *r_e_* = 50%; (**d**) *r_e_* = 25%.

**Figure 16:**
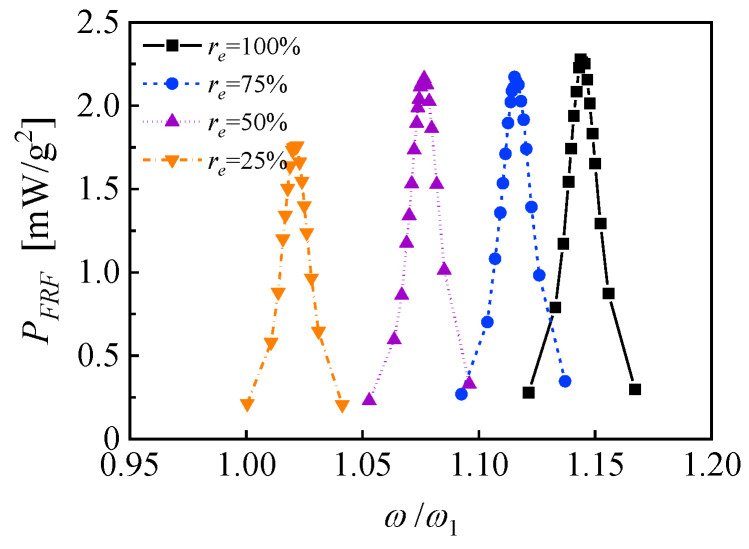
The characteristic power response curves obtained by coupling tuning for the unimorph cantilever based on PZN-PT.

The output power density FRFs of the PZN-PT-based PEH are also experimentally evaluated and shown in Figure 15. The base acceleration of the exciting vibration is set to 0.1 m/s^2^ to avoid the fragility of PZN-PT. When the EEC is 100%, the NOCR optimal load resistance is 14 MΩ, the NOCR frequency bandwidth is 1.74%, the frequency shift between the NOCR pick and NSCR pick is 14.4%, and the maximum power density FRF is 2.28 mW/g^2^. When the EEC is 25%, the NOCR optimal load resistance is 6 MΩ, the NOCR frequency bandwidth is 1.91%, the frequency shift between NOCR pick and NSCR pick is 2.1%, and the maximum power density FRF is 1.94 mW/g^2^. When the EEC is 75% and 50%, the frequency shift between NOCR pick and NSCR picks are 11.4% and 7.5%, and the maximum power density FRFs are 2.14 mW/g^2^ and 2.15 mW/g^2^, respectively. The differences among maximum power density FRFs may be attributed to the piezoelectric property variations contributed by the uncontrolled spontaneous nucleation and non-uniform crystallization orientation of the single crystal [29]. The output power density FRFs of the PEH in four EEC on the same load resistance of 14 MΩ are shown in Figure 16. The discrete distribution of the response curves indicates that a continuous frequency range of high-power response could be obtained by more sophisticated subdivisions of the electrode and the corresponding switching of EECs. According to the linear interpolations of the power response curves of EECs of 25% and 75%, a maximum potential frequency bandwidth of more than 14% can be obtained, which is improved by more than 700% compared to the bandwidth of the response of an independent EEC. It is predictable that the frequency bandwidth can be further broadened by upgrading the unimorph cantilever to the bimorph one and optimizing the structures.

## 5. Conclusions

This work proposes a new coupling resonance frequency tuning mechanism that can adjust the near-open-circuit resonance frequency by changing the effective electrode coverage of a piezoelectric cantilever. The linear model of a bimorph piezoelectric cantilever illustrates that the near-open-circuit resonance frequency is positively related to the coupling strength and can be adjusted by the effective electrode coverage. Analytical and experimental results support the increased bandwidth and the average power among bandwidth range through the coupling frequency tuning method. Adopting extraordinary piezoelectric single crystals and optimizing the proof-to-mass ratio and piezoelectric layer thickness ratio are effective approaches to improve the potential bandwidth. According to the theoretical analysis, a built-up frequency bandwidth of 71.9% can be obtained.

## Figures and Tables

**Table 1:** The individual and built-up frequency bandwidths and effective average power of the piezoelectric cantilever with various EEC and at the load resistance of 245.6 kΩ.

*r_e_*	10%	20%	40%	75%	Built-Up
Maximum power [μW]	192.3	191.2	192.1	182.1	192.3
Bandwidth [%]	2	2.1	1.9	1.5	4.9
Effective average power [μW]	148.8	149.1	150.2	144.7	164.4

**Table 2:** The near-open-circuit resonance responses of the PEHs based on PMN-PT and PZN-PT were obtained from unimodal solution and multi-modal solution.

	PMN-PT		PZN-PT	
	Unimodal	Multi-Modal	Unimodal	Multi-Modal
${\bar{\omega }}_{\mathrm{noc}}$	1.117	1.112	1.705	1.536
*P_max_* [μW]	295.8	297.9	282.5	285.9
*R_opt_* [MΩ]	0.4411	0.4000	4.162	2.433

## Data Availability

The data presented in this study are available on request from the corresponding author. The data are not publicly available due to privacy.

## Appendix A

The *r*th-order effective modal inertia force is given by

(A1) $${\bar{M}}_{r}=\left[m+M_{t}\delta \left(x-L\right)+\frac{1}{2}\frac{d\delta \left(x-L\right)}{dx}M_{t}L_{M}\right]\int_{0}^{L}\phi_{r}dx$$

where *δ*(*x*) is the Dirac delta function, *m* represents the mass per unit length of the bimorph, *M_t_* is the mass of the proof mass, and *L_M_* is the length of the proof mass.

The equivalent capacitance of the effective piezoelectric units is given by

(A2) $$C_{p}=2bL_{e}\varepsilon_{33}^{S}\varepsilon_{0}/h_{p}$$

where $\varepsilon_{33}^{S}$ is the relative permittivity component of the piezoelectric layer at constant strain, and *ε*_0_ is the permittivity component of the vacuum.

**Table A1:** Structure and material parameters of the bimorph cantilever.

Symbol	Description	Value
*b*	Width of beam	10 mm
*L*	Length of beam	30 mm
*h_s_*	Thickness of substrate	200 μm
*h_p_*	Thickness of PZT	200 μm
*L_M_*	Length of mass	10 mm
*h_M_*	Height of mass	1.8 mm
*ρ_s_*	Density of steel	7732 kg/m^3^
*ρ* *_M_*	Density of copper	8960 kg/m^3^
*ρ* *_p_*	Density of PZT	7018 kg/m^3^
$c_{11}^{s}$	Young’s modulus of steel	200 GPa
$c_{11}^{p}$	Young’s modulus of PZT	54 GPa
$\varepsilon_{33}^{S}$	Relative permittivity constant	3928
*e*_31_	Piezoelectric constant	−14.58 C/m^2^

**Table A2:** Properties of piezoelectric materials.

	PZT	PMN-PT	PZN-PT
$\mathrm{Relative}\;\mathrm{permittivity}\;\mathrm{constant}\;\varepsilon_{33}^{T}$	4373	6080	6099
Coupling factor *K*_31_	0.4	0.62	
Coupling factor *K*_32_			0.877
Piezoelectric constant *d*_31_ [pC/N]	−270	−920	
Piezoelectric constant *d*_32_ [pC/N]			−1346
$\mathrm{Elastic}\;\mathrm{constant}\;c_{11}^{p}$ [GPa]	54	19.8	
$\mathrm{Elastic}\;\mathrm{constant}\;c_{22}^{p}$ [GPa]			22.93
Density [kg/m^3^]	7018	7986	8333
